# “It’s Another Feather in My Hat”-Exploring Factors Influencing the Adoption of Apps With People Living With Dementia

**DOI:** 10.1177/14713012231185283

**Published:** 2023-06-26

**Authors:** Aoife Conway, Assumpta Ryan, Deirdre Harkin, Claire Mc Cauley

**Affiliations:** School of Nursing and Paramedic Science, 2596 Ulster University, Co Londonderry, NI, UK

**Keywords:** mobile applications, dementia, technology acceptance, technology adoption, assistive technology, apps, digital technology, focus group

## Abstract

**Introduction:**

With the growing interest and availability of mobile applications (apps) for people living with dementia, it is desirable to have a broader insight into how technology adoption may be further improved. This paper aims to explore the factors influencing adoption of mobile applications for people living with dementia.

**Methods:**

The recruitment of participants was facilitated through a dementia advocacy group of people living with dementia. A focus group design was applied to elicit discussion and to explore divergent views on the topic. The data was analysed using thematic analysis.

**Findings:**

The 15 individuals who participated in this study comprised of seven women and eight men within the age range of 60-90 years. This study reports key findings pertaining to the views and experiences of using mobile apps. Data analysis revealed the following four distinct themes; (Theme 1: Living with dementia)-“That’s the difficulty there even with apps or anything else.” (Theme 2: Motivation)- “It makes me feel good. I feel a little with it [laughs], that its not all gone in there” (Theme 3: Fears and Concerns)- “Can somebody else get into your personal memories?” (Theme 4- Support)– “So it’s important that we have that support”. Together these themes encapsulate the most influential aspects, as highlighted by the participants influencing the acceptance and adoption of apps.

**Conclusion:**

This paper explores the barriers and facilitators to app acceptance and adoption. This includes the importance of “feel good moments” and positive experiences, challenges associated with living with dementia, the importance of ongoing support, and security of the user’s information. This study adds to what is already known by capturing the views and experiences of people living with dementia in relation to the factors influencing the adoption of apps.

## Introduction

The prevalence of dementia is rising due to many factors including population growth and an ageing population (Nichols et al, 2022). With no disease modifying treatments available, the World Health Organisation (WHO) have presented a Global Action Plan on the Public Health Response to Dementia (WHO 2017). This plan emphasises developing effective, low cost, and contextually appropriate strategies to provide person-centred, gender-sensitive, culturally appropriate support for people living with dementia and their families and carers. Non-pharmacological interventions for the symptoms of dementia are appealing as they can provide useful, versatile approaches, with limited side effects and improve outcomes for people living with dementia (Yorozuya et al, 2019, Chalfont et al, 2020). There is a growing interest in mobile applications (apps) as a way of providing non-pharmacological interventions and much-needed support for people living with dementia and their carers (Lenca et al, 2017; Dunham et al, 2021). Previous studies have suggested that mobile apps on smartphones or tablets, can be a valuable resource for people living with dementia, as they provide opportunity to deliver interventions in a person’s own home, involving meaningful activities such as music (Cunningham et al, 2019), digital games (Ning et al, 2020), reminiscence (Ryan et al, 2020) and physical activity (Barisch-Fritz et al, 2020). The need for accessible, home-based interventions accelerated in 2019 during the coronavirus pandemic. At this time, to limit the spread of infections, most countries across the world imposed a variety of ’lockdown’ measures (Boutoleau-Bretonniere, 2020). Many people were confined to their homes and were permitted only to leave to purchase essential goods, partake in physical exercise, or seek medical input. Daily routines and activities had to quickly adapt to safeguard people living with dementia due to significant risk factors (Tam et al, 2021). Whilst lockdown measures dramatically changed most people’s daily lives, its impact may have been particularly pronounced for people living with dementia. Social distancing measures and diminished physical contact with family and the outside world, for many, resulted in increased loneliness and impacted mental health (LeVasseur, 2021; Fogelson, 2021). For some people, the only option for social connectedness was through the use of communication apps such as Zoom, Twitter, Skype, WhatsApp and FaceTime (Cuffaro et al, 2020; Gerritzen et al, 2023; Greenberg et al, 2020; Alhayan et al, 2023). However some studies suggest that although there was an increase in the engagement of apps designed for people living with dementia during the COVID-19 pandemic, this was not statistically significant (Kuo et al, 2022).

Although research suggests that mobile apps could significantly support people living with dementia and their carers, the rate of acceptance and adoption remains relatively low (Miguel Cruz et al, 2021; Øksnebjerg et al, 2020). Research in acceptance of technology has resulted in the development of several theoretical models that aim to understand and predict acceptance within multiple settings and across varied populations. These include (but are not limited to) the Technology Acceptance Model (Davis, 1989) and the Unified Theory of Acceptance and Use of Technology (UTAUT2) (Venkatesh et al, 2003, 2012). Despite such models, it has been argued that the understanding of user acceptance is limited. This is due to divergent interpretations of the concept, as well as inconsistent terminology used. Terms such as acceptability, acceptance, and adoption are often employed, sometimes interchangeably (Nadal et al, 2020). To address this Nadal et al. (2020) developed the Technology Acceptance Lifecycle (TAL), a continuum consolidating existing definitions (Nadal et al, 2020). The TAL consists of pre use acceptability; this C stage encompasses the period before any interaction with a technology occurs. The first interaction with a technology marks the end of pre-use stage and the beginning of the initial use acceptance stage. The final stage, sustained use acceptance, is when the user reaches the point of adopting the technology (Nadal et al, 2022). The TAL provides researchers with explicit terminology (as used throughout this paper), encourages terminology coherence, and articulates the different stages of technology acceptance. Current practices of reviewing adoption often focus the subset of acceptance factors as suggested by the aforementioned theories (Alam et al, 2018; Portz et al, 2019; Schretzlmaier et al, 2022). This means that other potentially relevant factors could be overlooked, reducing opportunities to improve the experience of using apps. For this reason, this study did not rigidly adhere to any specific theoretical framework but instead used an exploratory approach to gain a broader insight into the variables that influence technology adoption and abandonment for people living with dementia.

To explore what is already known about the factors influencing the adoption of digital health apps among people living with dementia, the authors undertook an integrative review of the literature (Conway et al, 2023). The review revealed a rich body of research surrounding technology adoption, however highlighted a dearth in the literature pertaining to factors impacting adoption of apps for people living with dementia. Although this informed the authors on what is already known, the authors felt the factors impacting adoption had not been sufficiently explored. There was a need to engage people living with dementia to capture their perspective, learn about their diverse lived experiences with mobile apps and consider their viewpoints. Furthermore, the steady growth in the use of mobile devices and mobile apps over the past years (both apps designed for people living with dementia and “off the shelf” apps) (Tangari et al, 2021; Haggag, 2022), provides a good opportunity to discuss these experiences with people living with dementia to provide a more detailed consideration of the factors that impact (enable or impede) technology adoption.

## Aim

The aim of the study was to explore, via focus groups, factors influencing adoption of mobile applications for people living with dementia.

### Objectives

(1) Understand the experience of using apps from the perspective of the person living with dementia.
(2) Explore the barriers and facilitators of using apps for people living with dementia.
(3) Consider how the experience of using apps could be enhanced.

## Methods

### Design

To conduct this study in a way that promoted a meaningful and active involvement with people living with dementia, a qualitative descriptive approach was applied and a focus-group design was selected. The focus group approach was chosen because of its ability to elicit discussion and to explore divergent views and interactions between group participants. Additionally, the synergy and spontaneity created by focus group discussions facilitates the sharing of experiences and voicing of opinions that may not surface during individual interviews (Hansen, 2020; Patton, 2002, Øksnebjerg et al, 2020). The research team discussed possible challenges and limitations associated with focus groups, such as how the group dynamics amongst participants could cause challenges. For example, the discussion being dominated by one vocal participant or participants engaging in side talk which could potentially hinder the opportunity to capture diverse perspectives (Kornbulah, 2022). To minimise these challenges, detailed field notes were taken to document engagement among all group participants. The researcher used a flexible approach to the discussion, to facilitate an authentic and engaged dialogue and to accurately capture a diversity of views and opinions of all involved (Roulston, 2010; Edwards & Holland, 2013; Nyumba et al, 2018). The facilitation skills of the research team were of vital importance in ensuring an enjoyable experience for the participants and overcoming any challenges. The researchers attended additional workshops on qualitative interviewing skills.

Focus groups have been widely used in health research in recent years to explore the perspectives of patients and other groups in the health care system (Santesso et al, 2022; George et al, 2022; Person et al, 2022). Focus groups have been previously used in dementia research (Bunn, 2015) however less widely in studies involving people living with dementia as participants in the focus group (Leverton, 2021). Studies that have adopted this approach have highlighted clear benefits including capturing non-verbal cues which may be particularly important when gathering information from people living with dementia. We followed the COREQ (see Table 1) to foster the transparency of the study (Tong et al, 2007).

**Table 1.** COREQ Checklist.

Topic	Item No.	Guide Questions/Description	Reported on Page No.
Domain 1: Research teamand reflexivity			
Personal characteristics			
Interviewer/facilitator	1	Which author/s conducted the interview or focus group?	7
Credentials	2	What were the researcher’s credentials? E.g. PhD, MD	Title page
Occupation	3	What was their occupation at the time of the study?	Title page
Gender	4	Was the researcher male or female?	Title page
Experience and training	5	What experience or training did the researcher have?	6
Relationship with participants			
Relationship established	6	Was a relationship established prior to study commencement?	6
Participant knowledge of the interviewer	7	What did the participants know about the researcher? e.g. personal goals, reasons for doing the research	6
Interviewer characteristics	8	What characteristics were reported about the inter viewer/facilitator e.g. Bias, assumptions, reasons and interests in the research topic	8
Domain 2: Study design			
Theoretical framework			
Methodological orientation and theory	9	What methodological orientation was stated to underpin the study? e.g. grounded theory, discourse analysis, ethnography, phenomenology, content analysis	
			4–5

Participant selection			
Sampling	10	How were participants selected? e.g. purposive, convenience, consecutive, snowball	
			6–7
Method of approach	11	How were participants approached? e.g. face-to-face, telephone, mail, email	6–7
Sample size	12	How many participants were in the study?	7
Non-participation	13	How many people refused to participate or dropped out? Reasons?	NA
Setting			
Setting of data collection	14	Where was the data collected? e.g. home, clinic, workplace	5
Presence of non-participants	15	Was anyone else present besides the participants and researchers?	
			5
Description of sample	16	What are the important characteristics of the sample? e.g. demographic data	Table 2
Data collection			
Interview guide	17	Were questions, prompts, guides provided by the authors? Was it pilot tested?	Table 3
Repeat interviews	18	Were repeat interviews carried out? If yes, how many?	NA
Audio/visual recording	19	Did the research use audio or visual recording to collect the data?	7
Field notes	20	Were field notes made during and/or after the inter view or focus group?	7
Duration	21	What was the duration of the interviews or focus group?	7
Data saturation	22	Was data saturation discussed?	7
Transcripts returned	23	Were transcripts returned to participants for comment and/or correction?	NA
Domain 3: Analysis and findings			
Data analysis			
Number of data coders	24	How many data coders coded the data?	7–8
Description of the coding tree	25	Did authors provide a description of the coding tree?	7–8
Derivation of themes	26	Were themes identified in advance or derived from the data?	7–8
Software	27	What software, if applicable, was used to manage the data?	7
Participant checking	28	Did participants provide feedback on the findings?	7
Reporting			
Quotations presented	29	Were participant quotations presented to illustrate the themes/findings? Was each quotation identified? e.g. participant number	9–19
Data and findings consistent	30	Was there consistency between the data presented and the findings?	9–19
Clarity of major themes	31	Were major themes clearly presented in the findings?	9–19
Clarity of minor themes	32	Is there a description of diverse cases or discussion of minor themes?	19–23

#### Ethical Considerations

This study was approved by University of Ulster Research Ethics Committee application number: REC/19/0098. The authors implemented safeguards to ensure all data was collected in an ethical manner. This study was designed and facilitated in collaboration with a partner organisation, a local independent voluntary organisation established by people living with dementia, driving positive change for people living with dementia. The organisation holds regular empowerment and support groups, facilitated by an empowerment officer, an employed member of staff (AD). Data was collected at naturally occurring empowerment and support groups held at their usual meeting space, a local community setting. On one occasion the researchers joined via zoom due to covid-19 restrictions, however the participants met as normal in their usual meeting space. The aim was to promote an infrastructure and environment that was conducive to the meaningful and effective involvement of people with dementia while also limiting disruptions and changes in established routines for participants. All participants had a well-developed, supportive relationship with the empowerment officer AD. The team ensured AD was available and willing to attend each focus group as a support to the participants. Carers of people living with dementia were also welcomed as a support for the participant, should they wish however only one participant availed of this. The data collection venues were assessed and deemed appropriate to enable each individual to contribute effectively and minimise the risk of any harm or distress occurring before, during or after the focus group (for example ease of access to toilets, sufficient light, signposting and space etc.). A Distress Protocol was developed, for implementation should any participant become distressed during the focus group, however this was not required at time of data collection. All members of the research team have a background in nursing or pharmacy and were experienced in communicating with and identifying the early signs of distress among people living with dementia. As this research was conducted with a pre-established group, the researchers took appropriate steps to understand group dynamics and to support each person to contribute meaningfully to the discussion (Gove et al, 2018).

The person living with dementia was provided with information about the research project and specific information on their proposed involvement that was clear and easy to understand (DEEP, 2013). Informed consent was provided by each participant who agreed to take part in the study with the additional clause of giving consent to use a digital recorder for the interviews. All participants were informed that they could withdraw their participation at any time. Assurances were provided about protection of anonymity and confidentiality, and this was supported by the allocation of pseudonyms in the presentation of the study and its findings (International Council of Nurses, 2021).

#### Recruitment and Participants

The recruitment of participants was facilitated through a dementia advocacy group of people living with dementia. The inclusion criteria specified (1) an adult living with dementia (2) aged 18 years or older (3) the ability to understand written and verbal information (4) willingness to provide subjective experiences and insights by engaging in a discussion (5) willingness to provide consent. The inclusion criteria did not have any requirement for participants to be currently using or have previously used an app. All members of this dementia advocacy group were monolingual removing language mixing or language reversion as a potential challenge.

Prior to the focus group, the Empowerment Officer (AD) explained the study and distributed a letter of invitation to potential participants who met the inclusion criteria. Interested participants were provided with additional information. This information was shared in advance, so the person living with dementia had time seek any necessary clarification or support. It was also important that they had a clear understanding of what the study entailed and their expected contribution prior to being asked to decide about any possible involvement. Contact details for the research team were provided so that participants who had any research-related questions were able to contact a member of the research team. Interested participants made themselves known to the Empowerment Officer (AD). A total of 15 individuals took part in the study.

#### Procedures and Data collection

A total of four focus groups were held. Number of participants in each group ranged from 2 to 5 and lasted approximately 30–40 min. On the day of each focus group, two members of the research team (AC and DH) provided an overview of the study, revisited the materials previously provided, reaffirmed consent, and answered any questions. Basic demographic data (age, sex, use of technology) were collected (see Table 2). The focus groups were supported by a semi-structured interview guide (Table 3). To ensure a positive experience for the participants there was flexible handling of the interview guide, whereby questions were rephrased, as required, to enhance clarity and encourage detailed responses. This helped to create a relaxed group atmosphere where participants were encouraged to talk freely about any aspect of the questions, they deemed important. At the end of each focus group, the researchers (AC and DH) drew upon the main points and asked participants if these represent their view and if they would like to add any points. Data collection was ceased when no new information regarding the aim of study was found from the focus group interviews. All discussions were recorded with the permission of participants. Both researchers fulfilled distinct roles during the focus group interviews, with one (AC) facilitating the focus group and the other (DH) obtaining observational data and field notes (Krueger and Casey, 2014). All data was collected between May/June 2022. Participants who were unable to attend or felt more comfortable in a one-to-one discussion were given the option of an individual semi-structured interviews, although no participants availed of this.

**Table 2.** Participant Characteristics.

Pseudonym	Group	Date	Age Range	Device used	Male/female
Susan	1	23.05.20	60–70	Tablet and smartphone	F
Richard	1	23.05.20	80–90	Computer, smartphone, laptop	M
Greta	2	10.06.22	70–80	Computer, tablet, smartphone, laptop	F
Seamus	2	10.06.22	70–80	Not currently using any devices	M
Mick	2	10.06.22	70–80	Smartphone	M
Greg	2	10.06.22	60–70	Computer, tablet, smartphone, laptop	M
George	3	17.06.22	60–70	Computer, tablet, smartphone, laptop	M
Valerie	3	17.06.22	60–70	Computer, tablet, smartphone	F
Helen	3	17.06.22	80–90	Computer, tablet, smartphone, laptop	F
Jim	3	17.06.22	70–80	Ipad, smartphone	M
Josie	3	17.06.22	70–80	Not currently using any devices	F
Tom	4	24.06.22	60–70	Tablet and smartphone	M
Brian	4	24.06.22	60–70	Smartphone, laptop	M
June	4	24.06.22	70–80	Ipad, laptop	F
Anna	4	24.06.22	80–90	Not currently using any devices	F

**Table 3.** Flexible Interview Guide.

Introductions and Welcome	• Thank you for agreeing to help us with this project • Explain study • Confirm consent • Inform participants that they can withdraw their participation at any time. • Provide assurances were provided about protection of anonymity and confidentiality • Answer any questions • Discuss group agreement
Questioning	1. Everyone’s experience of using apps is different. It would be helpful to hear of your experience of apps P: What do you use apps for? P: What do you most enjoy about using apps? P: What features of the apps make it easier/difficult for you to use? P: Have you had any problems or challenges using apps? P: How does using these types of apps make you feel? P: When you are not having a good day, would you use these apps to help you feel better or would you refrain from using them on these days? P: How often would you use apps (daily/weekly/monthly ect) If not currently using: P: Have you previously used apps? P: We know apps isn’t for everyone and that is ok. Is there anything in particular that stops you from using them? 2. We would really like to know what could be done to enhance your experience? P. What support would you find beneficial?
Closing	• Summarise main points • Confirm if main points these represent their views • Open the floor for any additional thoughts • Ask for any closing questions • Thank you

#### Data Analysis

Data from the focus groups were transcribed verbatim by an experienced transcriptionist and checked for accuracy. Microsoft Word 2016 was used for the management of data analysis. The data was analysed by AC according to Braun & Clarke’s (2006) thematic analysis method. The aim was to categorise and code the focus group data in a rigorous and systematic way and in doing so, to provide a comprehensive unbiased understanding of the topic. The findings are presented throughout the following sections. In direct quotations are used demonstrate evidence for the interpretations and to provide a greater depth of understanding. In vivo quotes were used to describe themes that best reflect the views of participants (Manning, 2017).

#### Credibility and Rigour

Several strategies were used to ensure rigor throughout the study (Lincoln and Guba, 1985). The focus groups were recorded and checked to ensure the rigour of the data collection procedures. Peer validation was completed with a third researcher (AR), all transcriptions were provided for AR to analyse. This provided a check on selective perception and illuminated any blind spots in the analysis. The goal of the peer validation process was not to reach a consensus, but to understand multiple ways of seeing the data, thereby enhancing credibility and rigour. The trustworthiness of the data was enhanced by the wider research team, who further reviewed themes maximising credibility, dependability, and confirmability (Lincoln and Guba, 1985). Measures such as reflexive discussions were used to minimise the influence of subjective perceptions, previous knowledge and personal bias on the study and on the analytical process (Whittemore et al, 2001, Varpio et al, 2017). A detailed description about the study context and rich information on findings (supported by excerpts from focus group interviews) which may enable the transfer of findings to similar socio-cultural context.

## Findings

The 15 individuals who participated in this study comprised seven women and eight men within the age range of 60–90 years. All participants explained they had previously used a device such as a smartphone, laptop, or tablet, however the frequency varied. Most of the individuals(*n* = 12) were using one or more devices, whilst the minority (*N* = 3) were not using these devices at the time of the study. The personal characteristics of individuals are outlined in (Table 2).

This study reports key findings pertaining to participants’ views and experiences of using mobile apps and factors that enable or impede adoption (Table 4).

**Table 4.** Overview of Barriers and Facilitators.

	Corresponding theme
Barriers to adoption	
Multifaceted changes associated with living with dementia	Theme 1: Living with dementia and using apps
Ability to use digital technology	Theme 1: Living with dementia and using apps
Reduced confidence	Theme 1: Living with dementia and using apps
Feelings of frustration and upset	Theme 1: Living with dementia and using apps
The person living with dementia did not see a purpose for the app	Theme 2: Motivation
Preference for a non-technology options	Theme 2: Motivation
Fear of making mistakes	Theme 3: Fears and concerns
The person living with dement had limited belief in their capacity and skills for integrating these technologies	Theme 3: Fears and concerns
The person living with dementia had a previous negative experience with using apps	Theme 3: Fears and concerns
Sharing of information to apps, particularly the sharing of personal and background information such as email addresses acted as a deterrent	Theme 3: Fears and concerns
Fears of over-sharing on the app due to diagnosis of dementia	Theme 3: Fears and concerns
The person living with dementia was not aware of the apps available to them	Theme 4-support
A paternalistic approach to support	Theme 4-support
The person living with dementia did not like to ask for help or assistance from others	Theme 4-support
Facilitators of adoption	
Support with use of the app (both initial and ongoing support)	Theme 1: Living with dementia and using apps/Theme 4-support
Support with app content	Theme 4-Support/Theme 3: Fears and concerns
Perceived usefulness	Theme 2: Motivation
Convenient nature of an app	Theme 2: Motivation
Functional and easy to use app	Theme 1: Living with dementia and using apps/Theme 2: Motivation
Societal influence	Theme 1: Living with dementia and using apps/Theme 2: Motivation
Apps which requested minimal personal information	Theme 3: Fears and concerns
Apps which highlighted the privacy-preserving precautions	Theme 3: Fears and concerns
A group or workshop that the person living with dementia could attend regularly for support	Theme 4-support
Repeated use and experience using the app	Theme 4-support

## (Theme 1: Living with dementia and using apps)–“That’s the difficulty there even with apps or anything else”

Several participants spoke of how living with dementia had an impact on their lives and they experienced changes in terms of their physical, social, emotional, and cognitive abilities. They discussed how these multifaceted changes made the challenge of using and adopting apps complex for people living with dementia:

“That’s the difficulty there even with apps or anything else. Everything that we do is just nearly overwhelming with difficulties and the app isn’t any different, and there’s some people can handle it and there’s some people that can't” (Brian, person living with dementia).

“Well I have had somebody help me with it and to be quite honest, from one day to the next it just seems to change. I just don’t seem to have taken to it. I’m not receptive to it. I can’t keep it in” (Jim, person living with dementia).

“It’s a process that you always need to keep practising that because it can fade away, it would fade away from me” (Seamus, person living with dementia).

Some people discussed how their ability to use digital technology had changed whilst living with dementia and this impacted their confidence in themselves and their abilities:

“Whenever I got dementia everything I done at work I was using Smart Phones and stuff like that there but see once I got dementia I lost it. Even to do simple things. I would struggle” (Tom, Person living with Dementia).

“One thing about dementia is loss of confidence. Most people with dementia will tell you that they’ve lost their confidence in one way or another and it’s very hard to restore and very hard to get back again” (Brian, person living with dementia).

Many participants questioned their judgment and felt a loss of control:

“It’s scary, you just suddenly think oh, what’s happening to me” (Greg, person living with dementia).

They explained how a loss of confidence had an impacted their willingness to try new things as well as undertaking tasks that they previously felt competent in. Some participants explained how if they had support, they would feel more at ease using apps.

“I feel safer when he’s in the house to use it with me.” (Susan, person living with dementia).

When the person living with dementia had difficulties with the app it often caused feelings of frustration and upset, for many participants these challenges were overwhelming and resulted in non usage:

“ I end up feeling a bit stupid, you know, to be quite honest like” (Jim, Person living with dementia).

“When I lost it, I just sort of walk away from it” (Brian, person living with dementia)

## (Theme 2: Motivation)- “It makes me feel good. I feel a little with it [laughs]. That its not all gone in there”

Motivating factors that influenced the intention or willingness to use an app were closely associated with positive experiences. Many participants specified that their motivation to use an app was often influenced by their belief that the app adds value to their day-to-day life (perceived usefulness).

“I’m looking for a purpose. (…) I need to have a purpose”(Richard, person living with dementia)

“You have to have the need for the app first otherwise what’s the point? What you’re looking at is what do I need to run me today” (Brian, person living with dementia)

Several perceived benefits were highlighted by participants including staying touch with family members, especially in times of isolation during the COVID19 pandemic. Using apps in this way facilitated communication and interaction between a person living with dementia and their carers, families, or friends. It also made an impact on how the person living with dementia felt. The participants report several “feel good moments” when using apps to communicate with others.

“It’s good just being in touch with them like, you feel that they’re there, just to see them you know, it’s unbelievable” (Helen, person living with dementia)

“Oh it was brilliant, particularly the tiny one. (…) so what age would she have been in lockdown, three? So you know, a phone call wouldn’t have worked . It was lovely to be able to still see her do her antics and do her wee dance and her cartwheels. So it was lovely. It made me feel like I wasn’t missing out as much, during lockdown” (Valerie, person living with dementia)

Whilst some participants remarked that being able to communicate in this way was beneficial and a positive experience others explained they felt digitally isolated from their friends or family as they could not communicate using this medium. As a result, these individuals did not see a purpose for the app which resulted in non-usage and impacted adoption.

“we have quite a big family and they sent a wee message yesterday every one of them and they’re from all over the world probably and they all said a wee note for me on the phone to say Happy Birthday …but I couldn’t answer them back. I couldn’t even say…I got the message... I couldn’t respond. And they’re probably sitting saying sure he didn’t even say anything or let them know I got it. I couldn’t respond back to them. (…) It’s just frustrating.” (Tom, person living with dementia)

Other participants explained how living with dementia can be incredibly lonely, and found that engaging with an app helped to prevent boredom and provided them with a meaningful activity other than watching the television:

“Aye. You can only watch so much TV. I get bored and lose interest. It’s like groundhog day. It’s terrible, just waiting to clock it.(…) now If I’m sitting doing nothing I can maybe lift it (points at phone)..Its a lifeline” (Seamus, person living with dementia).

Some participants suggested that a significant motivating factor was the convenient nature of an app in meeting their needs.

“Uhm I enjoy the convenience of being able to sit down press a button and it’s there immediately for me.” (Richard, person living with dementia)

Other benefits highlighted by the participants included engaging in reminiscence using an app. Some participants explained how it could be useful to use a mobile app to connect with memories. One person living with dementia explained that due to her diagnosis of dementia and the nature of the progressive illness, she felt it was important to store her memories so she could reminisce on these important times of her life if she deteriorated.

“I was thinking it would be a good thing to do because if more memories disappear, I would at least have prompts. I would call it a prompt box. I do collect bits and pieces. When I look back on bits and pieces of work I’ve done or been asked to do I’ve learned more from those incidents than what people told me. I don’t want to forget those prompts” (Greta, person living with dementia).

Some participants highlighted the importance of useablity, if an app that was functional and easy to use, this would motivate them to use the app:

“ As long as what’s on the app I can keep it simple. It’s keeping it simple and keeping it workable for me but it’s not that easy being workable for me. I feel sometimes it can be overwhelming.” (Brian, person living with dementia)

When participants spoke of apps that were easy to use, this was strongly associated with enjoyment, as well as sense of achievement and fulfilment felt by learning the new skill of using digital technology and apps. Many participants commented on how this was a significant personal achievement for them at this stage in their dementia journey. They explained that although they faced challenges with using mobile apps, it was worth the reward they experienced “feel good moments” which motivated them to continue to use an app.

“Uhm it makes me feel that I can do everything I do before, you know. ” (Richard, person living with dementia)

“It makes me feel good. I feel a little with it [laughs]. That its not all gone in there” (Helen, person living with dementia)

“I felt to myself I’ve actually achieved that, you know, it’s another goal for me, for a guy that has been ten years with dementia. It’s another feather in my hat that I can do that” (Brian, person living with dementia)

Some participants suggested that a societal influence had an impact adoption. Users often discussed how they were influenced by like-minded people or other people living with dementia to use apps and if others perceived the app as useful, this influenced the person living with dementias intention to use the app.

“Well I mean, its essential so you know, so you either join in or get left behind. Its like everything else is going on in the world.” (Richard, person living with dementia).

Whilst others suggested this acted as a deterrent and was off putting for the user. Some participants explained that reasons why others perceived the app as useful did not appeal to them:

“There’s some people there use it for everything and really, there’s some nonsense on it” (Seamus, person living with dementia)

“Well that’s another thing, to be quite honest. I’ve heard people talking about (…) and I think why would you want the bother of it” (Jim, person living with dementia)

In the case of participants who have not adopted apps or felt this type of medium was not appropriate for them they shared that they were not motivated to use apps as they preferred non-technology options and suggested that there would be no requirement to use an app if it did not add a benefit to their day-to-day life, impeding adoption.

“I’m not interested. As long as I’ve got my knitting or my crocheting or something.” (Josie, person living with dementia)

“I have a confession…. I hate mobile phones” (June, person living with dementia)

## (Theme 3: Fears and Concerns)- “Can somebody else get into your personal memories?”

Participants were keen to discuss their experiences and personal views of using apps, it became clear that fears and concerns played a significant role in app adoption. For example, some participants highlighted a fear of making mistakes, which in turn resulted in non usage:

“It’s nerves I think a lot of the way, you know, Im very anxious in doing it. I’m always frightened of making mistakes.” (Helen, person living with dementia)

“I’m very happy not doing it at all if I think I am going to mess up.” (Greta, person living with dementia)

Some participants shared limited belief in their capacity and skills for integrating these technologies. The relationship between these beliefs and motivation to use an app is clear as often these beliefs negatively influenced the intention to adopt apps:

“I’m a slow learner” (Jim, person living with dementia)

“I’m not that smart.” (Josie, person living with dementia)

“I’m useless with technology, absolutely useless. I’m useless with technology.”(Mick, person living with dementia)

Whilst others explained that their fears or concerns centred around the content of the app. When discussing an app for reminiscence, participants explained they had fears about remembering previous life events and they have memories they would prefer not to revisit:

“I’m afraid to reminisce… Bring up all sorts of things. I’ve been around the world many, many times and I’ve probably left a lot behind that could stay there.” (Brian, person living with dementia)

Other participants explained they had previous negative experiences with apps and this would deter them from using again.

“I’ve not had a lot of happy experiences with apps.” (Jim, person living with dementia)

Some participants shared their experience of receiving negative comments online, or being the target of scammers or fraud:

“The thing that frightens me about Facebook is there’s some ejits out there and that would be what would make me wary about what I would sort of post or even comment on because as I say, you get some people who would be very abusive back to you, you know, you put what you think is an innocent sort of statement and somebody can come back and make you feel upset .” (Valerie, person living with dementia)

For some participants, the simplicity of some apps and login features caused alarm. One participant explained features that have been designed to make some apps more accessible (such as fingerprint access/touchID) can cause alarm and can deter individuals from using apps.

“And he just said press on that link. And I only had that to press it but I thought well, if I only have that to do who else has that to do to get in. It’s okay to have it set up, that stupid old nana can just press a button to get what you want. But I’d like to know can everybody else press a button “ (Greta, person living with dementia)

Other concerns shared by participants included the sharing of information to apps, particularly the sharing of personal and background information such as email addresses

“Whenever you enter your email how secure is that. (all agree). I don’t like doing that for some reason, that’s just me. Where it would progress me to, what problem will it have.” (Helen, person living with dementia).

One participant also raised concerns about sharing her memories using an app for reminiscence and the risk of others accessing her precious memories.

“Can somebody else get into your personal memories? (…) I’ve always been very cautious because I wouldn’t want anybody using that to their advantage to involve me in anything. I’ve always worked with…my work was with people who were very traumatised and I would never have had information on my computer. . I stay away from that computer things like that because I’m worried I don’t know enough about the security.” (Greta, person living with dementia)

She explained that she dealt with many confidential matters throughout her career and had fears of over-sharing this information due to her diagnosis of dementia,

“And I’m very careful with who knows what I want. And who knows what I know. I have to be careful with what I know. And with the dementia sometimes that is difficult.” (Greta, person living with dementia)

Whilst some participants commented on how they preferred apps which required minimal personal information:

“I was impressed with you know, the fact that I was allowed not to put too many details on” (Greta, person living with dementia).

Other participants spoke about how they took privacy-preserving precautions on social media apps such as being careful about what information they post on and where they share information:

“I would be wary about what I would sort of post or even comment on…. I like our own enclosed Facebook groups because we put something on that and it’s safe and secure and some of the staff are sort of okaying it before it goes up and it’s sort of okay but even putting general sort of posts up” (Valerie, person living with dementia).

For majority of participants, for them to feel safe to use the app, it was important to know who could access their information. Some participants explained this is more than just a name on a screen, they need to trust the people they are sharing their personal information with; may this be app developers or people reading their posts on a social media app:

“You ring him because you know and you’ve laid eyes on him. But with them computer things you don’t know.” (Anna, person living with dementia).

## (Theme 4-Support)– “so it’s important that we have that support”

Throughout the focus groups many participants expressed an interest in wanting to learn or enhance their skills in digital technology, they expressed how they saw the value in using the apps to support daily activities. They discussed some of the aforementioned barriers with regards to using the technology and explained how they would require support to overcome some of these barriers.

“I’d feel like its (referring to using an app for reminiscence) something that’s gonna help me. I will do it if it helps me. But I need help. I cant do it on my own” (Jim, person living with dementia)

The participants highlighted an information gap, they explained that often they were not aware of the apps available to them particularly apps designed for people living with dementia, or how they would access apps:

“I probably wouldn’t know where to look for them…. so I’d need to be pointed in the right direction” (Tom, person living with dementia).

Majority of participants explained that they would require additional support to download and set up the app,

“So my family has set that up for me, so it’s important that we have that support.” (Brian, person living with dementia)

“I think I would need help to sit down and get it up and going” (Tom, person living with dementia)

Whilst initial support was valued, participants discussed how they required ongoing support when using an app, they explained that may need more explanation and a longer learning time, due to symptoms associated with dementia. Some participants explained how they would find value in a group or workshop that they could attend regularly for support with digital technology:

“I don’t know why I can't but it just seems so difficult and it’s stuff like that there…you know, it’s as if you would need a course every week.” (Tom, person living with dementia).

“I think if the workshop is always there, so if you had the workshop always there you’ve got it at hand, if you know what I mean. So yeah. It need to be continuous.” (Brian, person living with dementia).

The participants explained that the more they used the apps, the easier it became and often they required encouragement to use the app again.

“I really think if I was away from it for a few days I would lose all that ability so it’s crucial that people like me are encouraged to keep at it.” (Brian, person living with dementia).

Whilst majority of the participants voiced the need for support, at times they explained that loved ones can do elements *for* them rather than explaining or demonstrating how to do a certain task on the app:

“Mine think I can do nothing right so they don’t let me do it. Like I have to laugh at them, you know, I think they just think …granny, I’ll have to do that for her and I don’t enlighten them because it’s good to get it done sometimes” (Greta, person living with dementia)

“If I’m stuck my two daughters would just come in and push a couple of buttons and that would be it.. Done” (Mick, person living with dementia)

Whilst some participants found value in this approach others explained that given time and patience they could learn and develop their skills using apps and as previously mentioned this can bring a great sense of achievement:

“Have patience, especially with dementia. And once you do it again and again it does get easier you just need to stick with it. Have patience is what I say” (Mick, person living with dementia)

Some individuals raised concerns regarding their reliance on others to carry out tasks using apps and voiced worries of what would happen if their loved one was not available or present at a time they would need to use an app.

“Well my husband just does mine but I really should do it. I do get sort of very confused and I don’t sort of go out shopping and that on my own really but I sort of wonder what would happen if my husband wasn’t there and I wasn’t able to use the banking app but I think I would find it really confusing” (Valerie, person with dementia)

Additionally, some participants explained how it was not only the processes of using the app and support with the technology. They also expressed the need for support to enhance experience of using an app and with the content of the app. Participants often spoke positively about these episodes of support and engaging with app content. They associated this time with joy and happiness. Tom (person living with dementia) explained how using apps to reminisce on old memories with his children promotes communication and relationship building:

“Sometimes very sad. If you’re listening to music the music brings me back, especially playing the music when you were okay, everything comes drifting back and sometimes you sit and cry your eyes out. My kids would sit with me – you must remember this, da. When you were wearing the jeans turned up [laughs]. I have photos of it. The music brings you right back.” (Tom, person living with dementia)

The participants shared how they would avail of this support. For each person it was different. Some suggested support from family and friends, carers, local dementia charities and help groups:

“I had to get somebody to set me up, the grandson.” (Greta, person living with dementia)

“Well, (name of local support group) made it very simple for us to set up,” (Mick, person living with dementia)

Whilst majority of participants highlighted a clear need for support, some participants explained that they did not like to ask for help or assistance from others, often they would try again later at times this resulted in non usage.

“I don’t like doing that. Its not me. I don’t like …I don’t like…asking for help” (June, person living with dementia)

“Well, in my case I like to try to be as independent as I possibly can and asking for help is probably a let down for me, you know, so I’d rather grin and bear it ….most times in silence” (Brian, person living with dementia)

## Discussion

It is evident from the findings that the population of people living with dementia who are using apps are a diverse user group. The variables that influence acceptance and adoption are plentiful. Throughout the focus groups participants explained the diverse and divergent lived experiences of those living with dementia as well as the variation in capacities, needs, and preferences.

In keeping with findings by several other authors, (Hoque and Sorwar, 2017; Ma et al, 2021; Philippi et al, 2021) participants in this study explained how they would have increased motivation to use and accept an app if they perceived that it was more advantageous and meaningful in their daily life. The meaning ascribed to specific activities and reasons for participating in them varied between individuals. This highlights the need for a person-centred approach to identify apps that are meaningful and purposeful to support people living with dementia (Tierney & Beattie, 2020). Models of technology acceptance suggest the perceived usefulness of a technology influences the users’ intention to use and adopt (Davis, 1989; Venkatesh et al, 2012). However some participants were aware of the apps available, and the potential usefulness, but not interested in using apps. Contributing factors included a preference for non-technology options, and concerns about using technology (particularly in relation to fears and security) outweighed the possible benefits of using an app and therefore they decided not to use an app.

Participants voiced caution about using apps that they did not trust and raised concerns about their security and privacy. Given recent news coverage about cyber-attacks, fraud, exploitation as well as risk to security (Rane et al, 2022; Kajwang et al, 2022) it is not surprising that some participants had concerns about the uploading of sensitive or personal information. Participants felt uneasy that there was a lack of control over the exposure of their information and content. This had a strong influence on acceptance. With regards to “off the shelf apps” there are limited actions that can be taken to make this a safe environment for people living with dementia. However, with “bespoke” apps, there is an ongoing strive for regulators to provide governance within this area (Duggal et al, 2018). In this context, it is important to note that digital health management platforms such as the Organisation for the Review of Health and Care Apps (ORCHA) (https://orchahealth.com/) that assess apps against safety standards are an important resource and have the potential to provide much needed assurances to people living with dementia and their carers.

Another frequent concern highlighted within the literature is the cost associated with downloading apps. No participants in the current study discussed this as a factor influencing adoption (Venkatesh et al, 2012). It is a possibility that this factor was overlooked as all apps the participants discussed were free to download at the time of the study. A scoping review exploring adoption of Information and Communication Technology (ICT) for older people with chronic disease reported that despite successful outcomes, participants were unwilling to continue technology interventions that attracted a fee. In addition, when a technology intervention was perceived as expensive and complex, they lost interest (Zaman et al, 2022). Therefore, although not highlighted in the current study, cost may be a significant factor in adoption. Further research is required to explore this.

Throughout the study, participants provided an insight into how their level of cognitive impairment hindered their ability to recall information that was required to use an app. Some participants explained that over time, with frequent exposure to an app, they became more familiar with it and were able to use it with greater ease. This is in keeping with previous studies, who found with frequent exposure to an app over time people living with dementia reported increased confidence (Ryan et al, 2020) competence (Ekstrom et al, 2017; Ryan et al, 2020; Tyack et al, 2017), independence (Bielsten et al, 2020) and a newfound passion for technology (Critten and Kucirkova, 2019). McAllister et al, (2017) suggested that a health app should be introduced to a person with dementia as early as possible to support various aspects of their personal life as they could become familiar with the technology over time. Other studies acknowledge that people are more likely to engage with technology if they have learned how to use it before the onset of dementia. They suggest that if the person living with dementia can use an app in an easy intuitive way based on our prior usage, then he or she is more likely to utilise the technology (Naumann et al, 2011; Tyack and Camic, 2017). Despite this, a study by Astell et al, (2016) evaluating the concept of familiarity in gameplay found that familiarity is not a significant factor on its own and stresses the importance of apps that are enjoyable and easy to use. Within the current study participants were not asked if they had been using certain apps prior to a diagnosis of dementia, this is a possible limitation of this study, however, could be addressed in future studies.

In keeping with previous studies (Ma et al, 2021) participants in this current study suggested that ease of use is important when using an app. Although participants were not asked to identify features that impact the usability off apps, participants explained that, overall they preferred apps that were simple to use. The literature suggests that users usually feel connected to technologies that are convenient and simple to use (Ryan et al., 2020; Critten and Kucirkova, 2019; McAllister et al., 2017). The need for an inclusive design that addresses useability to reduce the level of challenge for people living with dementia is well documented within the literature (Gibson et al, 2016). The degree of usability is extremely important as this will significantly influence the user´s experience, which in turn will impact their level of motivation to continue using the app (Wildenbos et al, 2019).

The value of carers support when using an app is widely recognised within the literature (Critten and Kucirkova, 2019; Gall et al, 2020; McAllister et al, 2020; Rai et al, 2021; Ryan et al, 2020; Tyack et al, 2017; Øksnebjerg et al, 2020). However due to the large variation in people’s needs, the support users require differs from person to person. Some participants explained that they required support with knowing what apps were available to them, their benefits and how to access them, suggesting the need for effecting signposting to relevant and appropriate apps for people living with dementia.

Throughout this study participants highlighted a few occasions when the use of an app was influenced by other people. There was a consensus that if they did not use mobile apps they would get “left behind” and miss valuable experiences such as staying connected with friends and family. They were also concerned that they would not be kept up to date with valuable information, suggesting an external pressure to use apps. Sometimes this influence was explicit in that a relative or close friend directly suggested that a participant would benefit from using a specific app. Social influences also contributed to some participants’ reluctance to use apps. For example, a non-user stated that the reason they did not use social media apps was because a close family member had spoken unfavourably about an app and shared of negative experiences using the app. Bixter et al. (2019) reported a similar finding when studying older adults’ perspectives on social communication technologies, such as email and as social networking sites (e.g., Facebook, Instagram). They also suggested that age may play a factor as participants discussed that they did not know anyone else their age who used the Instagram app. Similarly in the current study some participants suggested that their carers held a perception that apps are for the younger generations and often underestimated their abilities to use an app. It appears that these attitudes towards technology create “digital divide” impeding adoption (Irazoki et al, 2021; Diaz-Orueta et al, 2020). This is a challenge as Øksnebjerg et al. (2020) found that if carers perceived an app as beneficial, they were more likely to encourage a relative with dementia to use it increasing likelihood of adoption. This highlights the need to challenge the perception and attitude towards the use of digital devices for people living with dementia and provide information on the how apps have the potential tools to improve their health and wellbeing.

Living with dementia had an impact on participants’ lives and they experienced changes in terms of memory, thinking, language, orientation, behaviour, and the capacity to perform daily activities. As a result, this largely impacted the persons judgement and confidence of trying new things-such as trying out an app. The findings suggest a strong link been self-efficacy and adoption intentions. With regards to technology adoption, the impact on self-efficacy is well reported (Sun et al, 2013; Ryan et al, 2020). Many participants discussed their beliefs regarding the technical skills, abilities or knowledge required to use mobile apps. A positive or negative association with these skills had an impact on adoption intentions, for example if a person had limited trust or belief in their own technical abilities, they were less likely to consider adopting an app. However, the belief of limited skills in this area did not deter some participants, instead they expressed a willingness to develop their skills and highlighted the need for support. A study by Mitzner et al. (2019) also found that a positive attitude toward using technology by older adults is associated with long-term adoption.

The requirement for ongoing support and education, and not just support at the initial set-up or download stages was also identified. Some participants suggested the idea of a workshops, where participants could learn together, whilst also supporting each other. This need for support and education has been discussed by others, however often there is an emphasis on support in the initial stages of use (Kerkhof et al, 2016). Other studies suggest the need for longer term support however the feasibility of long-term support in a sustainable way is unclear (Meiland, 2017). Some of the participants expressed the need and want to learn the skills with digital technology however their family members or carers “ do it for” them, suggesting a paternalistic approach to support (Smebye et al, 2015). This caused concern for some people living with dementia as they felt if a day came where they needed to use the app independently, they would not have the skills available to do so. Participants suggested the need for support that is autonomist and promoted their independence, where although they person living with dementia is asking for help, they are do not simply have a passive role. Support that is delivered, presented, and received in a manner that promotes confidence and empowers the individual is paramount.

## Conclusion

People are diverse and so are their reasons for the acceptance or abandonment of apps. This study engaged people living with dementia to capture their perspective, learn about their diverse lived experiences with mobile apps and consider their viewpoints on factors that influence adoption. Majority of the participants within this study expressed an interest in using apps and a desire to learn or enhance their skills in digital technology. The themes presented within this study (Theme 1: Living with dementia and using apps Theme 2: Motivation, Theme 3: Fears and Concerns, Theme 4-Support) provide an insight into the barriers and facilitators of using apps for people living with dementia. This included the importance of “feel good moments” and positive experiences, challenges associated with living with dementia, the importance of ongoing support, and security of the user’s information. These findings highlight important considerations for practice and future research. The findings discussed should be considered as a prerequisite in designing an application for people living with dementia and throughout the continuum of adoption to reduce the risk of non-usage. In addition, engaging with people living with dementia as experts regarding their own experiences plays a crucial role in understanding design features that address useability and therefor adoption, highlighting the need to involve people living with dementia at each stage of app development and evaluation. Further research on how support can be delivered and received in a manner that is sustainable and empowers the individual would perhaps add to the rate of acceptance and adoption. Findings discussed are important for app developers, people living with dementia and their carers.

## Strengths and Limitations

A strength of using focus group method was the extent to which participants were able to interact in their reflections, often eliciting richer and more complex exploration of ideas as the discussions progressed (Patton, 2002). However, we cannot be sure that the same results would have been obtained from individual interviews. There may be a danger that some respondents may have given responses that they thought their group members wanted to hear (Sim & Waterfield, 2019). Despite the strengths of the study, there are still limitations with regards to the diversity of people with dementia represented in the study. The study was undertaken in one geographical region in the UK Also, the number of participants was relatively small. There is concern about the findings' transferability and generalizability to populations with various diverse demographic and socio-economic statuses. However, a thorough description of the study has been provided for the reader to assess the degree to which the results can be transferred to their specific setting or context (Korstjens & Moser, 2018). A limiting factor of this study is that as participants spoke of their experience of apps in general and not specific apps, therefore the complexity of the apps being discussed by the participants may be ambiguous throughout the study. Despite these limitations, the present findings add to the growing body of literature on technology adoption.
